# The Golgi-Localized γ-Ear-Containing ARF-Binding (GGA) Proteins Alter Amyloid-β Precursor Protein (APP) Processing through Interaction of Their GAE Domain with the Beta-Site APP Cleaving Enzyme 1 (BACE1)

**DOI:** 10.1371/journal.pone.0129047

**Published:** 2015-06-08

**Authors:** Bjoern von Einem, Anke Wahler, Tobias Schips, Alberto Serrano-Pozo, Christian Proepper, Tobias M. Boeckers, Angelika Rueck, Thomas Wirth, Bradley T. Hyman, Karin M. Danzer, Dietmar R. Thal, Christine A. F. von Arnim

**Affiliations:** 1 Institute of Neurology, Ulm University, Helmholtzstraße 8/1, 89081 Ulm, Germany; 2 Institute of Physiological Chemistry, Ulm University, Albert Einstein Allee 11, 89081, Ulm, Germany; 3 Massachusetts General Hospital Institute for Neurodegenerative Disease, Massachusetts General Hospital, Charlestown, Massachusetts, United States of America; 4 Institute of Anatomy and Cell Biology, Ulm University, Albert Einstein Allee 11, 89081, Ulm, Germany; 5 Core Facility Laser Microscopy, Ulm University, Albert Einstein Allee 11, 89081, Ulm, Germany; 6 Institute of Neurology, Ulm University, Albert Einstein Allee 11, 89081, Ulm, Germany; 7 Laboratory for Neuropathology—Institute of Pathology, Ulm University, Helmholtzstraße 8/1, 89081, Ulm, Germany; Nathan Kline Institute and New York University Langone Medical Center, UNITED STATES

## Abstract

Proteolytic processing of amyloid-β precursor protein (APP) by beta-site APP cleaving enzyme 1 (BACE1) is the initial step in the production of amyloid beta (Aβ), which accumulates in senile plaques in Alzheimer’s disease (AD). Essential for this cleavage is the transport and sorting of both proteins through endosomal/Golgi compartments. Golgi-localized γ-ear-containing ARF-binding (GGA) proteins have striking cargo-sorting functions in these pathways. Recently, GGA1 and GGA3 were shown to interact with BACE1, to be expressed in neurons, and to be decreased in AD brain, whereas little is known about GGA2. Since GGA1 impacts Aβ generation by confining APP to the Golgi and perinuclear compartments, we tested whether all GGAs modulate BACE1 and APP transport and processing. We observed decreased levels of secreted APP alpha (sAPPα), sAPPβ, and Aβ upon GGA overexpression, which could be reverted by knockdown. GGA-BACE1 co-immunoprecipitation was impaired upon GGA-GAE but not VHS domain deletion. Autoinhibition of the GGA1-VHS domain was irrelevant for BACE1 interaction. Our data suggest that all three GGAs affect APP processing via the GGA-GAE domain.

## Introduction

Cleavage of the amyloid-β precursor protein (APP) by type I transmembrane aspartyl protease β-site APP cleaving enzyme 1 (BACE1) is the first proteolytic step in the production of amyloid-β (Aβ) [1–4]. Aβ is the major component of senile plaques, one of the histopathological hallmarks in Alzheimer’s disease (AD). APP and BACE1 are sorted through the endoplasmic reticulum and trans-Golgi network (TGN) to the plasma membrane via the secretory pathway. Consistent with its optimal activity at low pH, BACE1 primarily localizes to the TGN and late endosomes [2]. For cleavage of APP, both proteins have to be internalized from the plasma membrane or transported directly from the TGN to these compartments. Whereas the NPXY motif is the predominant sorting signal in APP transport, BACE1 expresses a dileucine sorting motif (DISLL) [5] that belongs to the acidic cluster-dileucine (AC-LL) sorting signals. Mutation of LL to AA in the BACE1 DISLL motif leads to retention of BACE1 at the plasma membrane [6–8]. Besides the ability of these sorting signals to bind to the clathrin coat complex, proteins expressing these motifs are typically transported through a pathway directly connecting the TGN and the endosomal/lysosomal system [9–11].

**Fig 1:**
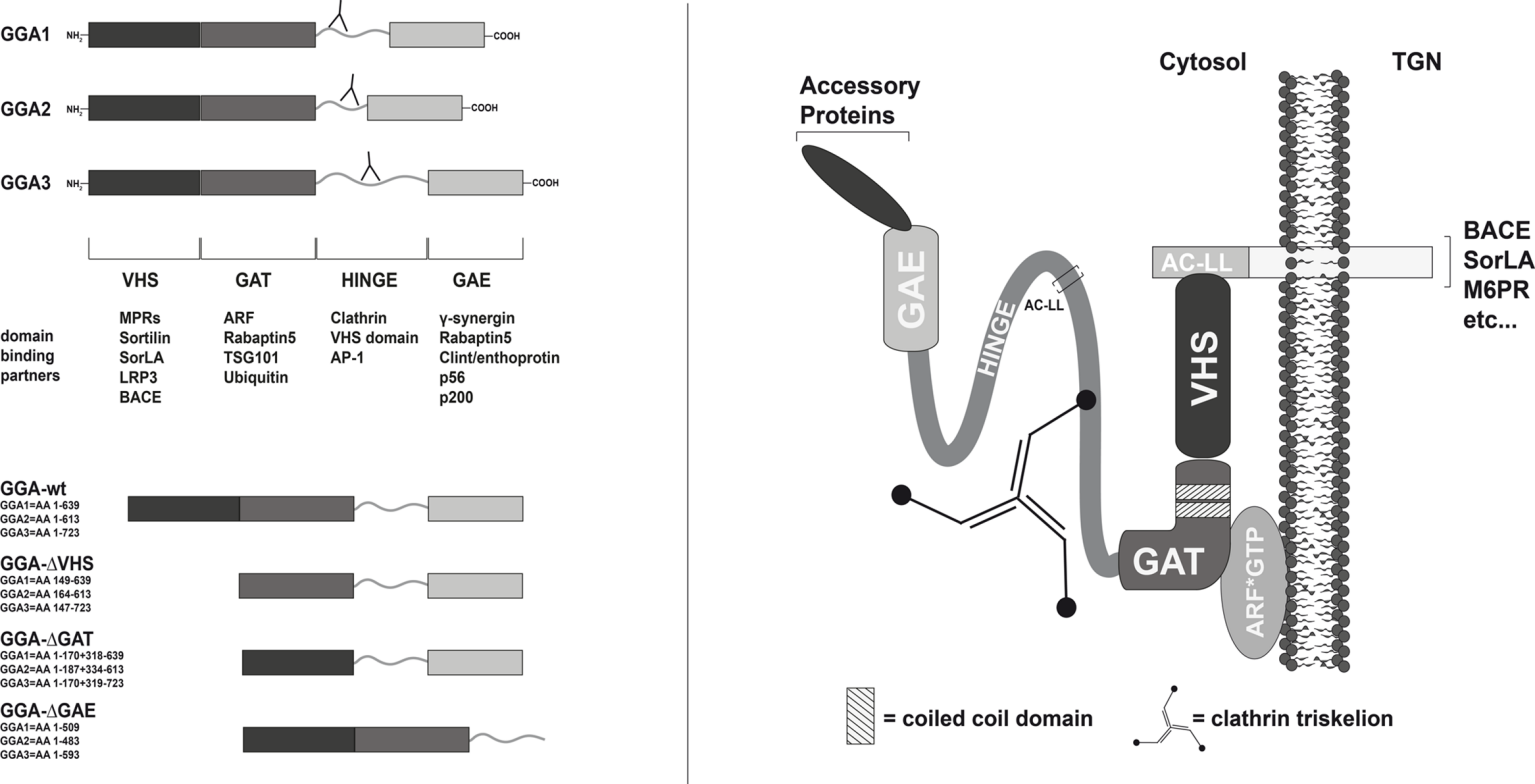
Human GGA protein family members. The GGA proteins were discovered almost simultaneously by five laboratories studying different aspects of membrane trafficking [14, 49–52]. The protein family consists of three members: GGA1, GGA2, and GGA3. GGAs are recruited from the cytosol onto the Golgi where they mediate the transport of cargo to endosomes/lysosomes. GGAs consist of four domains: the N-terminal VHS domain (Vps27, Hrs, and STAM); the GAT domain (GGA and Tom1), which contains two predicted coiled-coil domains; the hinge region, which contains one or more clathrin-binding sites; and the GAE domain (gamma-adaptin ear homology). The VHS domain is responsible for cargo recognition and binding via the AC-LL motifs in the cargo [6, 33, 53]. Membrane recruitment is provided by the GAT domain through ARF-GTP binding. Recently, the GAT C-terminal domains of GGA1 and GGA3 (but not GGA2) have also been shown to bind ubiquitin [54]. A distinct set of accessory proteins can bind to the GAE domain of GGAs, including rabaptin-5 [55], epsin R (Enthoprotin or Clint) [56–59], γ-synergin [14, 60], p56, and p200 [60]. Shown are the GGA deletion mutants used in this study (lower left panel). The antibodies used, directed against GGA1 (H215/Santa Cruz), GGA2 (BD), and GGA3 (BD), bind to the hinge regions of GGAs as indicated (upper left panel).

A family of proteins called the Golgi-localized γ-ear-containing ARF-binding (GGA) proteins was found to have striking functions in cargo sorting via their VHS domain and DXXLL motifs in this pathway [12, 13] (Fig 1). GGA proteins are monomeric in the cytosol and hence unlikely members of a multi-subunit adaptor complex [14]. However, studies have shown that the three GGAs interact with each other and co-localize within the same compartments [15]. Besides their function in cargo sorting at the TGN membrane, GGAs are thought to play an additional role in endosomal trafficking. Several studies provide evidence that GGAs can also shuttle ubiquitinated cargo to endosomal pathways via ubiquitin binding sites in their GAT and VHS domains [16–19]. GGAs were shown to transport cargo independently of DXXLL and VHS motifs [13, 20]. Moreover, BACE1 and GGA3 interaction was found to be independent of the DXXLL motif and VHS domain, supporting additional cargo binding sites and mechanisms [21]. Binding of the VHS domain of GGA1 and GGA3 to its cargo, in contrast to that of GGA2, requires dephosphorylation of a casein kinase 2 site within their hinge region. Phosphorylation of serine 355 in the hinge region of GGA1 and GGA3 has been proposed to lead to conformational changes and autoinhibition of the VHS domain by an AC-LL motif downstream of the casein kinase 2 site [22]. Furthermore, transport of BACE1 was shown to depend on phosphorylation of the serine residue in the DISLL motif by casein kinase 1. Whereas phosphorylated BACE1 crosses early endosomal compartments on its way to juxtanuclear structures, nonphosphorylatable mutants of BACE1 are retained in early endosome antigen 1-positive compartments [8, 23, 24]. In addition, phosphorylated BACE1 was shown to interact with GGA1 exclusively at juxtanuclear compartments [23]. Further study revealed that GGA1 also confines APP to the Golgi and perinuclear compartments and thereby impacts Aβ generation [25].

Since the expression of GGA1, 2, and 3 and their influence on APP processing have not been comparatively studied, we extended previous studies to compare the influence of GGA1, 2, and 3 on BACE1 interaction and APP processing. As there are several potential binding sites, we addressed the question of which domain interacts directly with BACE1. To determine which GGA isoforms are most pathophysiologically relevant, we analyzed GGA levels in rat, and human postmortem brain tissue. Specific altering of APP transport and processing by the modulation of GGA-mediated transport or sorting are promising targets that might alter the pathway leading to enhanced Aβ generation.

## Material and Methods

### Antibodies and Expression Constructs

For this study, the following primary antibodies were used: mouse (ms) anti-(α) APP N-terminus (22C11, Millipore/MAB348); rabbit (rb) α APP C-terminus (Sigma/A8717); rb α BACE1 (Calbiochem/195111); rb α GGA1 (H215, Santa Cruz/sc-30102); ms α GGA2 (BD/612612); ms α GGA3 (BD/612310); ms α c-myc (9E10, Sigma/M4439); rb α green fluorescent protein (GFP) (Sigma/G1544); ms α β-actin (AC-15, Sigma/A5441); ms α β-III-tubulin (Tuj1) (Covance/MMS-435P); rb α CD11b (Abcam/ab75476); rb α GFAP (Abcam/ab7779); and rb α hemagglutinin (HA) (Sigma/H6908). Secondary horseradish peroxidase or Alexa-dye coupled antibodies (Alexa-488, -647) were obtained from Molecular Probes/Invitrogen. The control vectors used were pEGFP-N3 (Clontech), pcDNA3.1-myc (Invitrogen), and phCMV3 (Genlantis).

APP-GFP, APP-mRFP, BACE1-GFP, SorLA/LR11-GFP, and GGA1-myc constructs have been described previously [23, 26–28]. BACE1-mRFP was generated by replacing the APP cDNA of the APP-mRFP construct with BACE1 cDNA of the BACE1-GFP constructs by using NheI and SacII cleavage sites. BACE1-HA was generated by subcloning BACE1 cDNA into the BamHI cleavage site of phCMV3 (Genlantis). BACE1-cterm-HA was generated by subcloning the cDNA of the BACE1 sorting signal and propeptide followed by the transmembrane domain and BACE1-cterm into phCMV3 (Genlantis). An untagged SorLA construct was generated by cutting EGFP cDNA out of the SorLA-GFP vector by using the flanking AgeI cleavage sites. A stop codon was inserted by using the Quick Change II site-directed mutagenesis kit (Stratagene, La Jolla, CA). EGFP cDNA was inserted between the propeptide and the Vps10p sequences of SorLA cDNA to generate the EGFP-SorLA construct. GGA2-myc and GGA3-myc were generated by using the same approach as described for GGA1-myc. GGA1, 2, and 3 cDNAs were cut out of the myc constructs at the NheI and XhoI sites and inserted into the pEGFP-N3 vector to generate GGA-GFP constructs. GGA1_S355A/D mutants were generated by using the Quick Change II site-directed mutagenesis kit (Stratagene, La Jolla, CA), with GGA1-GFP as template. To create deletion constructs, cDNA sequences of GGA-ΔVHS, GGA1-ΔGAT, and GGA-ΔGAE were cloned into pcDNA3.1-myc (Invitrogen, Carlsbad, CA) after amplification by PCR. The resulting amino acid compositions for all GGA deletion mutants are shown in Fig 1. Authenticity of the PCR-generated constructs was confirmed by DNA sequencing.

### Cell Culture and Transient Transfection of N2a and HEK293

Cells lines used in this study were obtained from Leibniz Institute DSMZ-German Collection of Microorganisms and Cell Cultures. Human embryonic kidney cells (HEK293) (DSMZ no.: ACC 305/ obtained 2008) and murine neuroblastoma Neuro-2a cells (N2a) (DSMZ no.: ACC 148/ obtained 2013) were cultured under standard cell culture conditions in Dulbecco’s Modified Eagle’s Medium (GIBCO) supplemented with 10% fetal calf serum and 1x penicillin/streptomycin. Transient transfections were performed 24 h after seeding with SatisFection (Stratagene) (N2a) according to the manufacturer’s instructions, the calcium phosphate transfection method (HEK293), or Lipofectamine 2000 (Invitrogen) (HEK293). Cells were incubated for 24 to 48 h prior to experiments.

### Protein Knockdown

HEK293 cells were transfected with FlexiTube GeneSolution siRNA (Qiagen) against GGA1, GGA2, and GGA3 alone or in combination by using Lipofectamine 2000 (Invitrogen). Experiments were performed 48 h after transfection. Protein knockdown was evaluated by Western blotting.

### Immunocytochemistry

HEK293 and N2a cells were grown on glass slides and transfected at low cell density. Twenty-four hours after transfection, cells were fixed in 4% paraformaldehyde/sucrose, permeabilized by 0.02% TritonX-100, and blocked with 1x Roti-ImmunoBlock (Roth) in phosphate-buffered saline (PBS).

Fluorescence images of immunostained cells were recorded with an Axiovert 200 microscope (Carl Zeiss, Jena, Germany). For further image analysis, files were processed with ImageJ (http://rsb.info.nih.gov/ij).

### LDS-PAGE, Western Blotting, and Detection

Cell samples were lysed in BEX buffer (25 mM Tris at pH 8.0, 20 mM NaCl, 0.6% w/v deoxycholate and 0.6% Igepal CA-630) and electrophoresed under denaturing conditions with NuPage Novex Bis-Tris 4%-12% gradient gels (Invitrogen) and MOPS running buffer (Invitrogen). Proteins were transferred semi-dry on polyvinylidene difluoride membranes and blocked in 1x RotiBlock (Roth) for 1 h. Incubation with primary antibodies was performed overnight at 4°C in 1x RotiBlock. The next day, blots were incubated with horseradish peroxidase-coupled secondary antibodies and analyzed with LAS-4000 (GE) by using ECL Luminata Forte (Millipore).

### Co-Immunoprecipitation (CoIP)

HEK293 cells grown in 100 mm dishes were transiently transfected with myc-tagged GGA-wild type (wt), -ΔVHS, -ΔGAT, or-ΔGAE mutants and BACE1-HA. Twenty-four hours after transfection, cells were washed with PBS and lysed in precooled lysis buffer (Miltenyi Biotec) on ice. Cell lysates were centrifuged, and supernatants were transferred to a new tube and incubated with anti-HA-coated MicroBeads (Miltenyi Biotec) for 1 h on ice. Labelled HA-tagged proteins were purified on MicroColumns (Miltenyi Biotec) following the manufacturer’s instructions. Proteins were eluted with 50 μl preheated (95°C) NuPAGE lithium dodecyl sulfate (LDS) sample buffer (Invitrogen) containing 100 mM dithiothreitol and analyzed by LDS-PAGE and Western blotting.

### In Vitro CoIP

For *in vitro* pulldown, HEK293 grown in 100 mm dishes were single transfected with the desired plasmids. Twenty-four hours after transfection, cells were lysed in 1 ml lysis buffer (Miltenyi Biotec). For magnetic labeling, supernatants were incubated on ice with anti-tag MicroBeads of choice for 1 h. Cell lysates were applied onto equilibrated MicroColumns, washed four times with high salt buffer (500 mM NaCl, 1% Igepal, 0.5% sodium deoxycholate, 0.1% sodium dodecyl sulfate [SDS], 50 mM Tris-HCl at pH 8.0), and washed two times with wash buffer 2. Proteins were eluted by nondenaturing elution of the column-bound antigen by using a pH shift according to the manufacturer’s instructions. Small samples of the eluates were analyzed by LDS-PAGE and Western blot to check for purification. For CoIP, eluates were combined, filled up to 1 ml with lysis buffer (Miltenyi Biotec), and incubated overnight at 4°C. The next day, magnetic labeling, purification, and elution were done as described earlier. Eluted immunoprecipitates were analyzed by SDS-PAGE and Western blot.

### Secreted APP Alpha/Beta (sAPPα/β) Measurements

N2a or HEK293 were transfected as indicated. After 24 h to 48 h of expression, media were collected. sAPPα and sAPPβ levels in the conditioned media were determined by sAPP Multiplex Assay kit (K15120E) and the SECTOR Imager 2400 (Mesoscale Discovery) following the manufacturer’s instructions.

### Aβ Measurements

N2a cells were transfected with APP695-GFP, BACE1-HA, and myc-tagged GGA-wt or GGA mutants as indicated. Aβ38, Aβ40, and Aβ42 levels were determined in cell culture supernatants by using Multi-Spot Human (6E10) Aβ Triplex Assay (K15148E) and the SECTOR Imager 2400 (Meso Scale Discovery) according to the manufacturer's instructions.

### Human Tissue

Human autopsy brain tissue was received from the Massachusetts Alzheimer Disease Research Center (MADRC, Charlestown, MA, USA) Brain Bank (#P50 AG005134-31) and the Ulm University Brain Bank (Ulm, Germany (Approval No.: 54/08)) in accordance with the laws and with the specific approval for this study of the local ethical committees. All experiments were performed in accordance with the Declaration of Helsinki. Clinical diagnosis was based on combined *Diagnostic and Statistical Manual of Mental Disorders* (4th ed.) criteria. For Western blot analysis, brain tissue was homogenized in BEX lysis buffer containing Halt phosphatase/protease inhibitor cocktail (Pierce). Protein (50 μg) was loaded onto 4%–12% Novex Bis-Tris gels and transferred to polyvinylidene difluoride membranes. Proteins were visualized by chemiluminescence. Clinical diagnoses of the patients of which brain tissue samples were used for Western blot analysis are shown in Table 1.

**Table 1:** 

Lane	Clinical Dx	Neuropath Dx	Age at death(y)	Gender	Dis.Duration(y)	APOE	PMI(h)	Plaque burden(%)	Braak	CERAD
1	AD	AD	84	F	15,54	3,4	12	4,318053	NA	C
2	CTRL	CTRL,HYPOXIA	89	F	0	NA	13	0	NA	NA
3	AD	AD	80	F	12	4,4	10,5	4,966679	NA	C
4	CTRL	CTRL	76	F	0	3,3	24	NA	I	No
5	AD	AD	86	M	16	3,4	12	3,051486	NA	C
6	CTRL	CTRL	56	M	0	3,4	36	1,66542	0	No
7	AD	AD	79	M	12,08	3,4	24	3,163545	VI	C
8	CTRL	CTRL	85	M	0	3,3	24	0	II	No

### RNA Extraction, cDNA Synthesis, and Quantitative PCR

Total RNA was isolated by using the mirVana isolation kit (Ambion) following the manufacturer's instructions. RNA concentration was measured with the NanoDrop (Peqlab) device. cDNA synthesis was performed with the Transcriptor High Fidelity kit (Roche) according to the manufacturer's instructions. A LightCycler 480 and Sybr Green (Qiagen) were used for quantitative RT-PCR. PCR reactions were performed in triplicate, with Rpl13 used as a reference for relative quantification. The results were corrected for primer efficiency, which was determined by serial dilution of a calibration cDNA mix.

### *In Situ* Hybridization

*In situ* hybridization was performed as previously described [29]. Tissue was obtained from 3 days (PD3), 9 days (PD9), 15 days (PD15), 21 days (PD21) and 5 month (adult) old male Spraque Dawley rats. BACE1 expression and GGA expression were detected by hybridization using specific anti-sense oligos purchased from MWG Biotec (Ebersberg, Germany):

GGA1 5’-GCCCACTTCGTGATGGAACCTCTTGCCACAGCTCTTCAT-3’,

GGA2 5’-GGATCCGGTCCCCGCCGCCACAGCCGTCGCTGCCAT-3’,

GGA3 5’-GGATTGGCCAGCAGCATGACCTGGGTGATGGCTGCAGGT-3’,

BACE1 5’-GCTGCCCACGGTCATCTCCACATAGTAGCCCTGGCCGGA-3’.

### Generation and Cultivation of Primary Cells

For the primary cell culture, C57BL/6J mice at embryonic day 18 (E18) were used. The preparation procedure was performed as described previously [30]. Briefly, pregnant mice were humanely killed according to the general guidelines for animal experimentation by cervical dislocation. The procedure was approved by the Institutional Animal Care and Use Committee of the Ulm University. E18 embryos were extracted and humanely killed by decapitation. Whole brains were removed from the skull, the hemispheres were separated, the meninges were removed, and the hippocampi were dissected from the remaining brain tissue. For dissociation, hippocampi were incubated with 2.5% trypsin and mechanically dissociated by pipetting. Cell count was determined and corrected to the desired cell density. Primary hippocampal neurons were maintained in Neurobasal media containing B27 supplement at 37°C, 5% CO_2_, and 10% O_2_. The medium was changed 45 min after plating.

Astrocytes and microglia were prepared as previously described [31]. Briefly, E18 embryos were extracted and humanely killed by decapitation. Whole brains were removed from the skull, hemispheres were separated, and meninges were removed. Brains were enzymatically dissociated by 1% trypsin and 0.05% DNase. The resulting cells were centrifuged, resuspended in Dulbecco’s Modified Eagle’s Medium containing 10% fetal calf serum and 1x penicillin/streptomycin, and plated into 75 cm^2^ flasks precoated with 1 μg/ml poly-L-lysine (Sigma). After 12–14 days in culture, floating and loosely attached microglial cells were manually shaken off and recovered onto cell culture dishes for cell lysis and Western blot analysis. Cells that remained attached were reincubated with cell culture media, and repopulating microglial cells were removed every week. After 5–6 weeks, attached astrocytes were rinsed once with PBS, detached, and centrifuged. Cells were plated onto cell culture dishes for cell lysis and Western blot analysis.

All experiments and procedures on animals in this specific study were approved and in accordance with the guidelines by FELASA and the Institutional Animal Care and Use Committee of Ulm University

## Results

### GGA Overexpression Decreases and Knockdown Increases sAPP Secretion in HEK293 and N2a Cells

**Fig 2:**
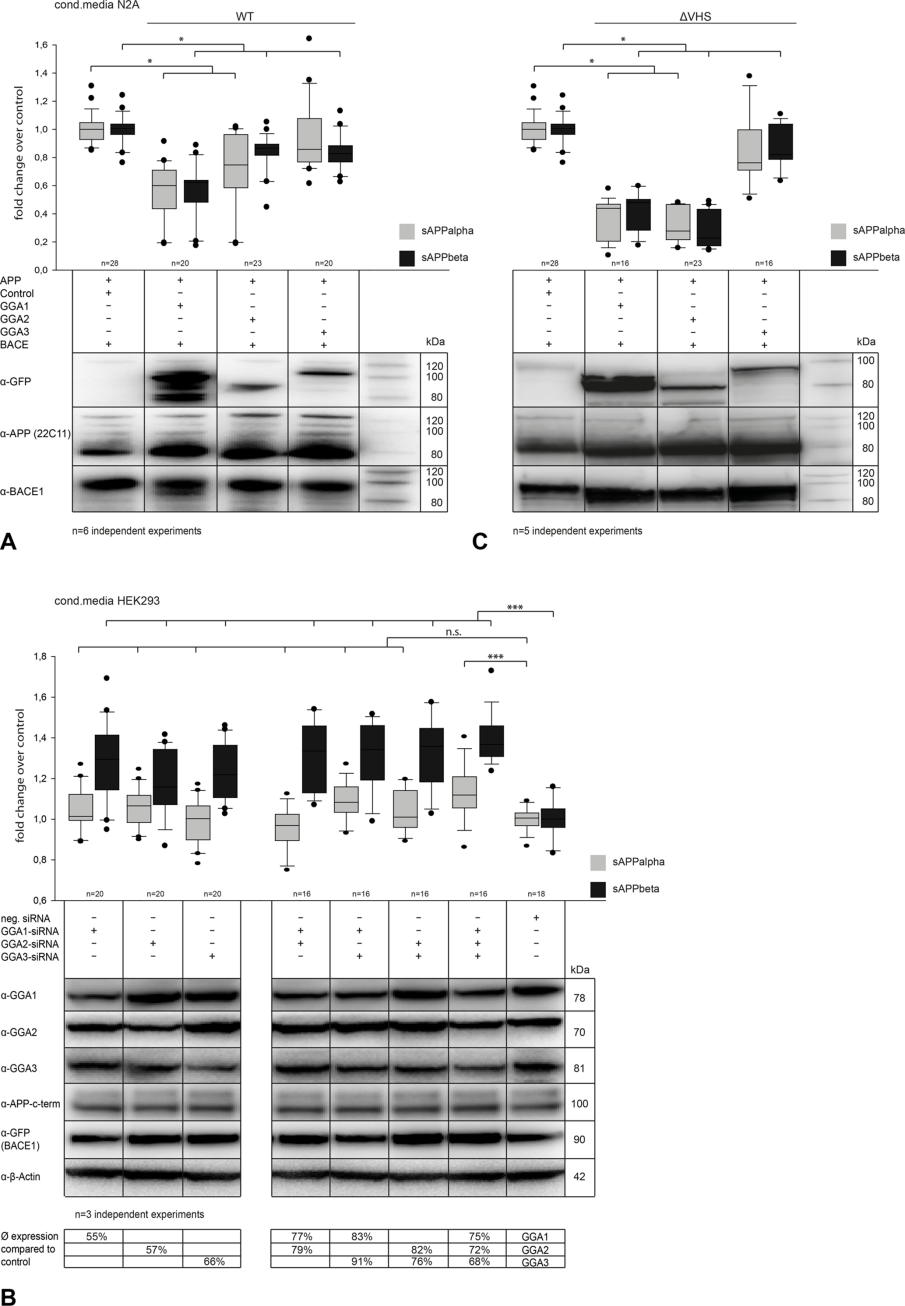
Regulation of GGA expression alters APP processing and sAPP secretion. (A) N2A cells were co-transfected with APP and BACE and either GGA1, 2, or 3. Equal expression of all proteins was controlled by Western blot analysis. We found, similar to the results in HEK293 (data not shown), significantly decreased levels of sAPPα and sAPPβ in conditioned media upon GGA overexpression compared with control, as measured by ELISA. Experiments were carried out in triplicate; shown are the results of n = 6 independent experiments. Statistical analysis was performed by using Kruskal-Wallis one-way analysis of variance (ANOVA) on ranks and multiple comparison (Dunn’s method) (*p<0.05). (B) HEK293 cells were transfected with FlexiTube siRNA (Qiagen) and incubated for 48 h. siRNA-mediated knockdown of single GGAs in HEK293 cells led to increased sAPPβ but not sAPPα levels compared with negative control. A combination of two or three GGA knockdowns led to an additive effect and stronger increase in sAPPβ secretion. Experiments were carried out in triplicate; shown are the results of n = 3 independent experiments. Statistical analysis was performed by using one-way ANOVA and Bonferroni t-test (*p<0.05; **p<0.01; ***p<0.001). (C) To analyze the influence of GGA-VHS domain deletion, N2A cells were transfected with APP-mRFP, BACE1-mRFP, and either GGA1-, GGA2-, or GGA3-ΔVHS-GFP. Deletion of GGA VHS domains did not lead to a reversed effect on sAPP secretion. Levels of sAPPs were decreased, similarly to the conditioned media of cells overexpressing wild-type (wt) GGA. Additionally, deletion of GGA2-VHS led to an even stronger decrease in sAPP levels. Experiments were carried out in triplicate; shown are the results of n = 5 independent experiments. Statistical analysis was performed by using Kruskal-Wallis ANOVA on ranks and multiple comparison (Dunn’s method) (*p<0.05).

Previous studies have reported that GGA1 and GGA3 affect APP processing and reduce secretion of sAPPα and sAPPβ. We here extended these studies by including GGA2 and performing a comparative study to test a possible functional redundancy of the different GGAs in APP metabolism. To do so, we first performed GGA1, 2, and 3 overexpression in N2a cells co-expressing APP and BACE1 (Fig 2A) and in HEK293 cells co-expressing BACE1 (data not shown) and analyzed conditioned media for altered sAPP levels. We found significantly reduced levels of sAPPα and sAPPβ for single GGA1, 2, and 3 overexpressing cells, as measured by enzyme-linked immunosorbent assay (ELISA) (Fig 2A). Furthermore, intracellular levels of sAPPβ were increased as shown by Western blot and ELISA assay (S1 Fig) Simultaneous expression of two or three GGAs had no additive effect on sAPP secretion (data not shown). To obtain more physiological conditions than in overexpression conditions that might also lead to impairment of transport mechanisms, we performed siRNA-mediated knockdown of GGAs in HEK293 cells (Fig 2B). Downregulation of single GGAs had the reverse effect on sAPPβ secretion but not on sAPPα. Simultaneous knockdown of two or all three GGAs further increased sAPPβ secretion.

### Processing of APP and Secretion of sAPPs Is Independent of VHS-DISLL Interaction

The fact that siRNA-mediated knockdown affects mainly sAPPβ levels indicates that the effect on APP processing is dependent on GGA-BACE1 interaction. To test whether we could change APP processing by altering VHS domain autoinhibition, we used GGA-nonphosphorylated or pseudophosphorylated mutants.

GGA1_S355A is a nonphosphorylatable and thereby constitutive active form of GGA1, whereas GGA1_S355D mimics the phosphorylated and autoinhibited form of GGA1. Using these mutants, we found no difference in the sAPP levels of GGA1_wt, GGA1_S355A, or GGA1_S355D overexpressing cells, as measured by Western blot analysis (S2A Fig). As GGA autoinhibition has recently been called into question [32], we next tested the idea that impaired sAPP secretion can be reversed by overexpression of GGAs lacking the complete VHS domain (ΔVHS). Surprisingly, sAPP secretion was also decreased compared to control (Fig 2C). Furthermore, intracellular levels were also increased, similar to those in GGA_wt and phospho-mutants (Fig 2C).

**Fig 3:**
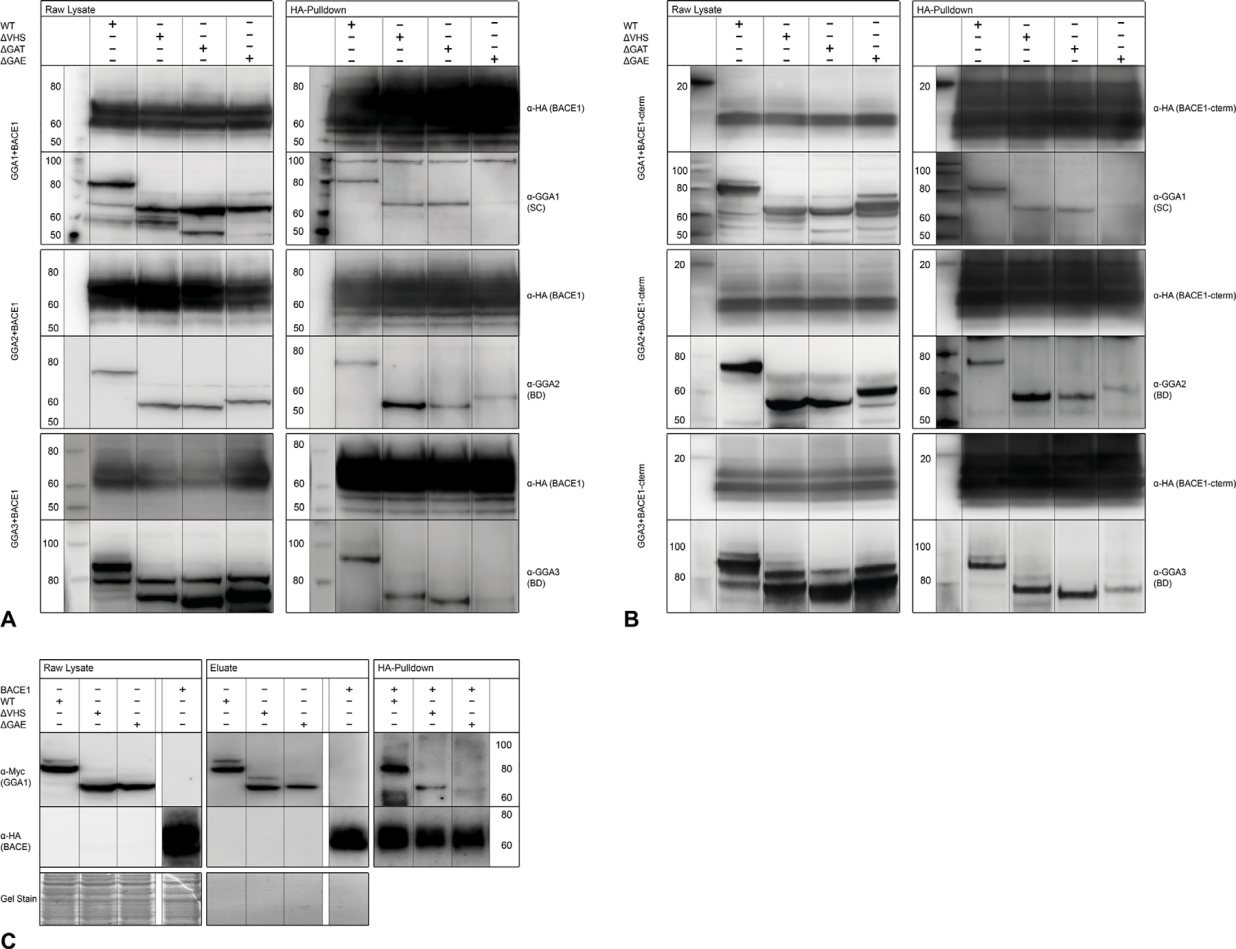
Deletion of GGA GAE domain but not VHS domain disrupts BACE1-GGA interaction in CoIPs. (A) HEK293 cells were co-expressed with BACE1-HA and either myc-tagged GGA_wt, -ΔVHS, -ΔGAT, or-ΔGAE constructs. Equal expression was controlled by Western blot (left panels). BACE1-HA was immunoprecipitated with anti-HA beads (Miltenyi Biotec), and precipitation and co-precipitation of the GGAs was controlled by Western blot (right panels). Deletion of the VHS domain of GGA 1, 2, or 3 did not disturb interaction with BACE1 compared with wt GGAs (Lanes 1+2). Likewise, deletion of the GAT domain had no effect on binding to BACE1 (Lane 3), whereas deletion of the GAE domain dramatically reduced binding of GGAs and BACE1 (Lane 4). (B) HEK293 cells were co-expressed with BACE1-cterm-HA and either myc-tagged GGA_wt, -ΔVHS, -ΔGAT, or-ΔGAE constructs. Equal expression was controlled by Western blot (left panels). BACE1-cterm-HA was immunoprecipitated with anti-HA beads (Miltenyi Biotec), and precipitation and co-precipitation of the GGAs was controlled by Western blot (right panels). Comparable to full-length BACE1, deletion of the VHS domain of GGA1, 2, or 3 did not disturb interaction with BACE1-cterm compared with wt GGAs (Lanes 1+2). Likewise, deletion of the GAT domain had no effect on binding to BACE1 (Lane 3), whereas deletion of the GAE domain dramatically reduced binding of GGAs and BACE1-cterm (Lane 4). (C) We tested whether BACE1 GGA1-GAE domain interaction is due to a direct binding of both proteins by using *in vitro* CoIP. Myc-tagged GGA1-wt, -ΔVHS, and-ΔGAE and HA-tagged BACE1 were expressed separately in HEK293 (left panel) and purified by immunoprecipitation using magnetic beads (Miltenyi Biotec) labeled with the corresponding antibodies (middle panel). Purified proteins were mixed and immunoprecipitated with anti-HA beads. Both GGA1-wt and GGA1-ΔVHS were co-precipitated by BACE1, but GGA1-ΔGAE was not, indicating direct binding of BACE1 and the GGA1-GAE domain.

Because neither GGA phosphorylation mutants nor VHS deletion alters functional APP processing and secretion, we next tested whether autoinhibition of GGA1 or ΔVHS mutants of all three GGAs impair BACE1-GGA interaction in CoIPs. In line with our previous experiments on APP processing, GGA co-precipitation was influenced neither by deletion of the VHS domain (Fig 3A) nor by autoinhibition of GGA1 (S2B Fig). It has been reported that VHS-DISLL interaction is functional only when the DISLL motif is located at the very C-term of the protein and that this interaction can be blocked by addition of more than two amino acids. For CoIP, we used a BACE1 with a C-terminal HA-tag. We observed no disturbed binding based on the HA-tag. In contrast to BACE1-GGA1 interaction, SorLA-GGA1 interaction was blocked by addition of a C-terminal tag (S3A Fig, S3B Fig). Furthermore, deletion of the GGA1 VHS domain impaired GGA1-SorLA interaction (S3C Fig). This further suggests that BACE1-GGA interaction is mediated by domains other than DXXLL-VHS interaction.

### Direct Interaction between BACE1 and GGAs Is Dependent on the GGA-GAE Domain and BACE1-Cterm

As recent data from other groups have also given rise to the idea of alternative interaction sites between BACE1 and GGAs [21], we created GGA deletion mutants lacking either the GAT or GAE domain (Fig 1). To exclude false positive results due to altered distribution and function of the mutants, all myc-tagged GGA variants were expressed in N2A cells, immunostained, and analyzed by fluorescence microscopy. As expected, deletion of the GAT domain of all three GGAs, which is responsible for membrane recruitment, let to an equal, nonphysiological distribution throughout the cell, whereas ΔVHS and ΔGAE mutants showed expression patterns similar to that in GGA_wt (S4 Fig). We next performed immunoprecipitation experiments by using lysates from HEK cells overexpressing BACE1 and either wt GGAs or one of the deletion mutants. Whereas GGA-ΔGAT mutants were co-precipitated to the same amount as GGA-wt and GGA-ΔVHS, co-precipitation of GGA-ΔGAE mutants was impaired for all three GGAs (Fig 3A), indicating that the GAE domain mediates GGA-BACE1 interaction. In contrast, interaction between SorLA and GGA1-ΔGAE was not altered compared with control (S3C Fig, right panel). To test whether this interaction is mediated by BACE1-cterm, we used a BACE1 deletion mutant consisting of the BACE1 transmembrane domain and the c-term only. We found the same binding pattern with the BACE1-cterm construct as observed with full-length BACE1 (Fig 3B).

To further test whether BACE1-GGA interaction is mediated by a protein complex or by direct binding between the GAE domain and BACE1, we performed *in vitro* CoIP. GGA1_wt-myc, -ΔVHS-myc, -ΔGAE-myc, and BACE1-HA were expressed separately in HEK293 cells (Fig 3C, left panel). Proteins were purified from cell lysates by immunoprecipitation according to their protein tag (Fig 3C, middle panel). Purity was controlled by gel staining. Purified BACE1-HA was mixed with either purified GGA1_wt, -ΔVHS, or-ΔGAE. Subsequently, BACE1-HA was again precipitated by HA beads and co-precipitation of GGAs was controlled by Western blot. Whereas GGA1_wt and GGA1-ΔVHS were detectable in Western blot analysis, GGA1-ΔGAE was not co-precipitated by BACE1, confirming direct binding between the GAE domain and BACE1 (Fig 3C, right panel).

### Deletion of GGA GAE Domain Increases Secretion of sAPPα sAPPβ and Aβ40 and 42

**Fig 4:**
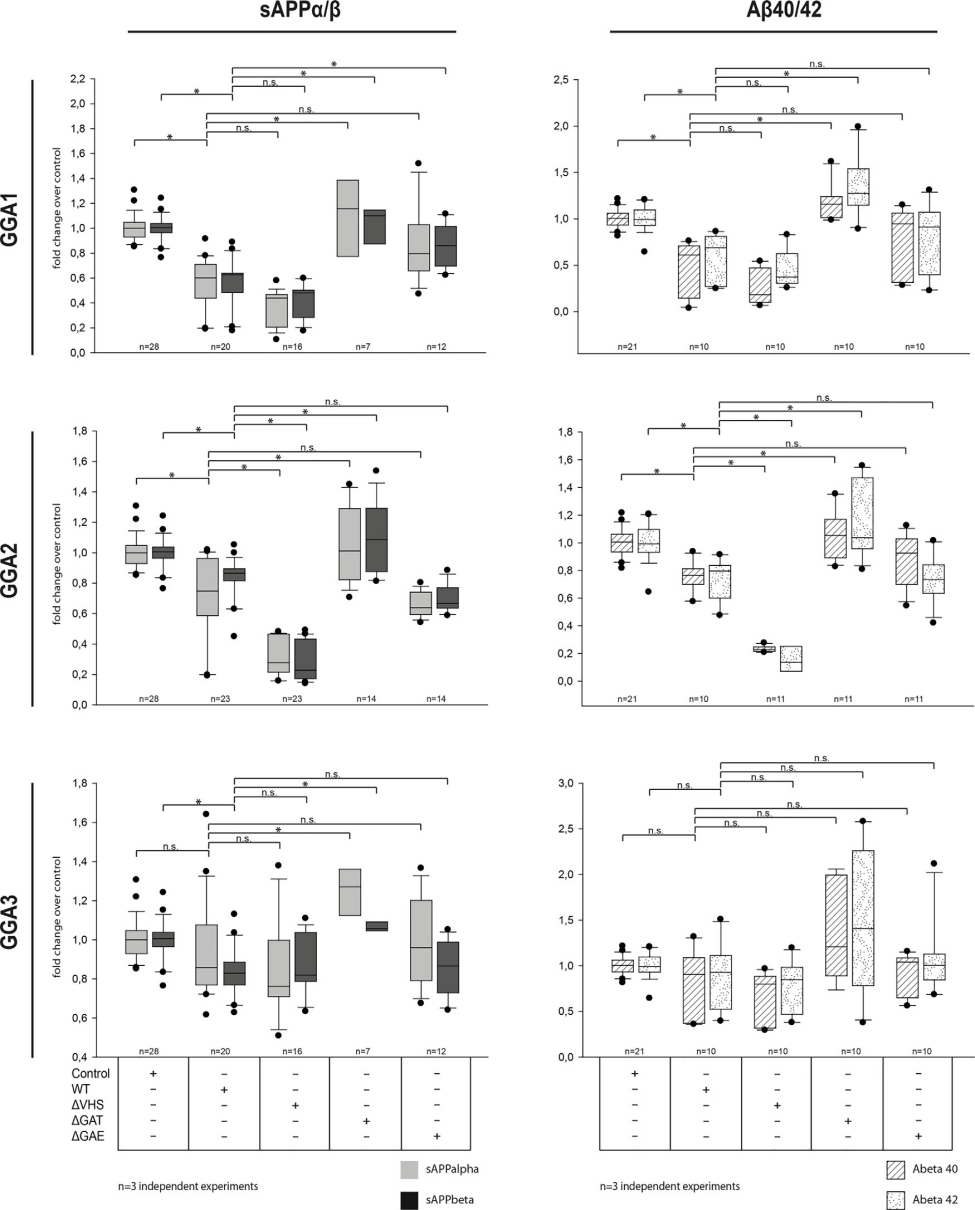
Deletion of GGA GAT and GAE but not VHS domain increases secretion of APP β-cleavage products. N2A cells were co-transfected with APP-GFP and BACE1-HA and either GGA1, 2, or 3_wt-myc or domain deletion mutants. After 24 h, sAPP and Aβ levels were measured in the conditioned media by using sAPP and Aβ ELISA kits (MesoscaleDiscovery). As shown previously, secretion of sAPPα and β were reduced in cells overexpressing GGA1, 2, or 3 _wt or-ΔVHS compared with controls (left panels). Additionally, Aβ40 and Aβ42 levels were also reduced in cells co-expressing GGA_wt or-ΔVHS. Deletion of the GGA-GAT domain completely abolished the effect of sAPPs as well as Aβ secretion. Deletion of the GGA1 GAE domain but not GGA2 or 3 significantly increased sAPPβ levels compared with that of GGA_wt. Likewise, Aβ levels were increased for all three GGA GAE mutants, indicating altered APP processing due to altered BACE1-GGA interaction. Experiments were carried out in triplicate; shown are the results of n = 3 independent experiments. Statistical analysis was performed by using Kruskal-Wallis ANOVA on ranks and multiple comparison (Dunn’s method) (*p<0.05).

Using ELISAs, we next analyzed the functional impact of GAT and GAE domain deletion on sAPP secretion compared with ΔVHS and GGA_wt. As shown before (Fig 2A and 2B), overexpression of GGA_wt and-ΔVHS decreased sAPP levels in the conditioned media (Fig 4, left panel). Deletion of the GGA2 VHS domain primarily had this effect, but was also seen for deletion of the GGA1 VHS domain. Likewise, secretion of Aβ40 and 42 was decreased upon overexpression of GGA1-, 2-, or 3-wt or ΔVHS mutants. In contrast, upon overexpression of GGA1-, 2-, or 3-ΔGAT sAPP and Aβ40 and 42, secretion was increased compared with GGA-wt but also with empty-vector control (Fig 4, left and right panels), most likely because of missing membrane recruitment of GGAs (S4 Fig). Deletion of the GGA1-GAE domain also increased sAPP and Aβ secretion compared with GGA1-wt. Upon GGA2-ΔGAE and GGA3-ΔGAE overexpression, particularly Aβ40 levels, were increased upon expression, similar to that in GGA1.

### GGA1, 2, and 3 Are Differentially Expressed and Regulated in the Brain

**Fig 5:**
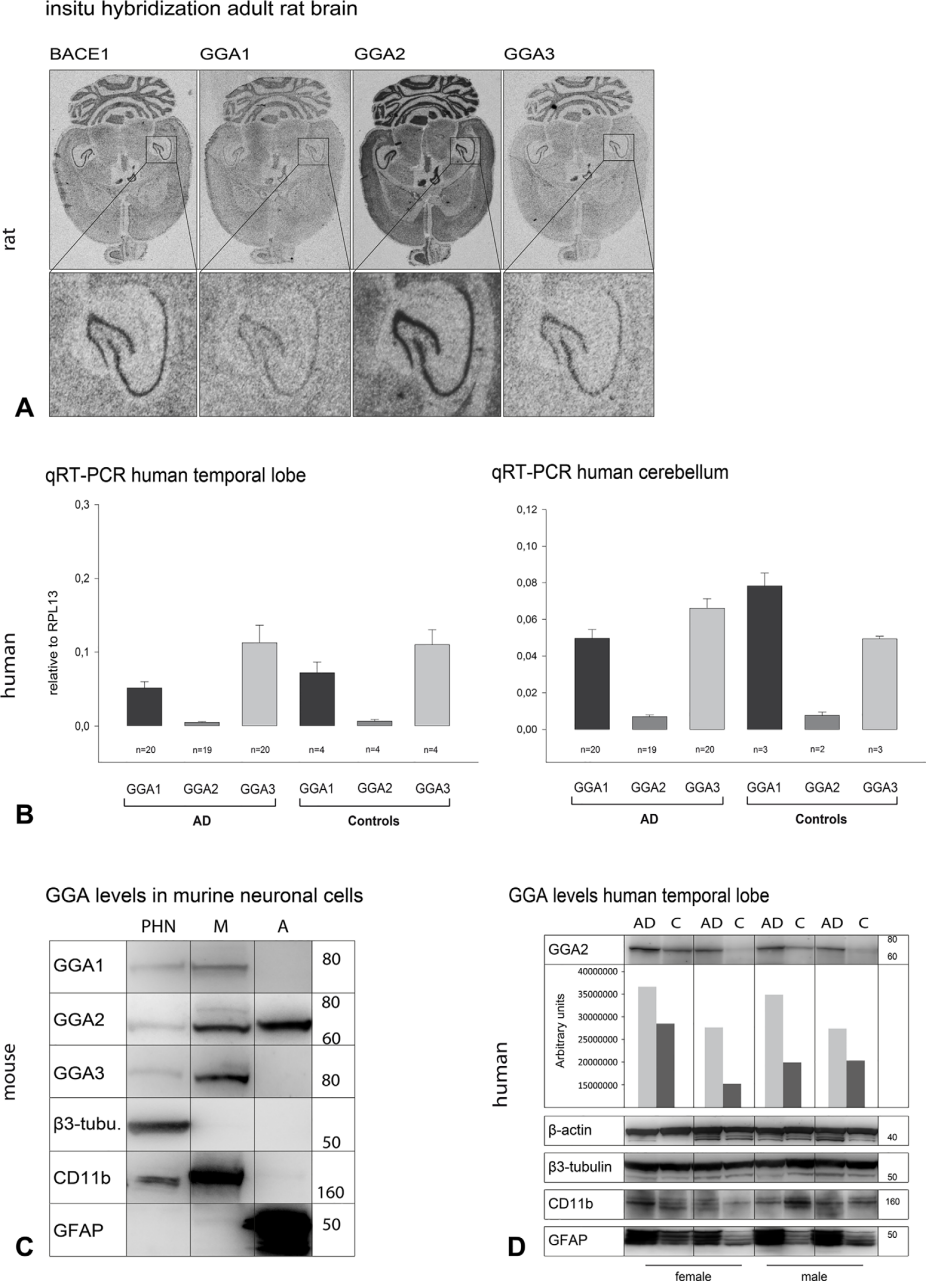
GGA1, 2, and 3 are differentially expressed and regulated in the brain. (A) *In situ* hybridization of subsequent horizontal sections of 5 month old male rat brain shows the neuronal expression of BACE1 as well as GGA1-3 in all brain areas investigated. The expression pattern of the genes is essentially identical with the highest mRNA levels in the granular cell layer of the cerebellum and within the hippocampal formation (see enlargement). A comparison of the mRNA levels of the different genes shows that GGA2 mRNA appears to have the highest mRNA concentration in brain. (B) mRNA levels of GGAs in human postmortem brain tissues were analyzed by qRT-PCR. We observed lower levels of GGA2 mRNA in temporal lobe and cerebellum samples as compared with GGA1 and 3 levels. No difference between controls and AD patients was observed either for GGA1, GGA3, or GGA2 levels. (C) Primary hippocampal neurons (PHN), microglia (M) and astrocytes (A) were obtained from E18.5 C57/Bl6 mice. GGA protein levels were analyzed by Western blot. Purity of the cell types were controlled by appropriate marker proteins. All three GGAs are expressed in PHN as well as microglia, but GGA2 was exclusively detectable in astrocytes. (D) GGA2 levels were analyzed by Western blot in postmortem temporal lobe samples of AD and control patients (MADRC). In samples with increased GGA2 levels, we also found increased levels of astrocytes, as indicated by increased GFAP levels. This finding points to a specific role of GGA2 in astrocytes.

As we observed that the three GGAs show changes in similar directions but yet can act synergistically, we analyzed whether GGAs and BACE1 are differentially expressed in brain tissue during development and adulthood to test their (patho-) physiological relevance in mammals. To do so, we performed *in situ* hybridization in brains of postnatal (S5 Fig) and adult rats (Fig 5A). BACE1 was found to be expressed in all brain regions, but was most prominent in thalamus, striatum, hippocampus, and granule cells. GGA1 showed ubiquitous expression in all brain regions, including the hippocampus. GGA2 seems to be more highly expressed in later stages of development. It showed high expression in the cerebellum, thalamus, and striatum, but was most dominant in the hippocampus. GGA3 was also expressed in later developmental stages and also showed expression in the cerebellum and hippocampus in adult animals. We found spatial- and time-correlated expression of GGA1, GGA2, GGA3, and BACE1 in rat hippocampus during postnatal stages and also in adult animals (Fig 5A). In the *in situ* experiment, GGA2 seemed to have higher expression compared with GGA1 and GGA3. Therefore, we next performed qPCR with mRNA obtained from human postmortem brain samples. We observed lower levels of GGA2 mRNA in temporal lobe and cerebellum samples compared with those of GGA1 and GGA3 (Fig 5B). However, we found no significant difference in expression between AD and controls. To further test differences in GGA expression pattern between different neuronal cell populations we used lysates of murine primary hippocampal neurons, microglia and astrocytes from E18 animals in western blot analysis. We found all three GGAs expressed in primary hippocampal neurons and in microglia (Fig 5C). However, in astrocytes from E18 animals only GGA2 was detectable. As recent studies have shown alterations of GGA1 and GGA3 protein levels in postmortem samples of AD patients, we also compared the expression of GGA2 in 26 temporal lobe samples obtained from control and AD patients. Some AD patients showed altered GGA2 levels compared with matched controls (Fig 5D). In the cases of increased GGA2, we also found increased levels of astroglial marker GFAP. This finding supports the idea of an additional role of GGA2 in astrocytes.

## Discussion

Since the discovery that APP cleavage by BACE1 is the rate-limiting step in Aβ production and that internalization and transport of both proteins to endosomes is crucial for this event, much effort has been put into preventing or modulating the co-occurrence of the two proteins in these compartments. We and others have recently demonstrated that GGA1, GGA2, and GGA3 can interact with BACE1 and thereby alter BACE1 subcellular distribution and APP processing [21, 23, 25, 33–35]. To further study the role of the different GGA family members in BACE1 activity, we analyzed APP processing and transport of its cleavage products in different cell lines.

We found that GGA overexpression resulted in decreased levels of secreted sAPPα and sAPPβ. To address the limitations of overexpression, we used knockdown of two or more GGAs simultaneously, which led to increased secretion (Fig 2B), primarily of sAPPβ. These results extend the findings of others that have shown that GGA3 knockdown leads to increased BACE1 levels in endosomes and enhanced activity and APP processing by BACE1 [21, 34, 36]. Immunoanalytical assays revealed an intracellular increase of sAPPs upon GGA overexpression (Fig 2, S1 Fig). We have previously reported that overexpression of GGA1-wt decreases levels of secreted sAPPβ and Aβ, while increasing sAPPβ and C99 fragments intracellularly [25]. One possible explanation is that GGA overexpression does not diminish APP processing by BACE1. Our data support the idea that GGAs do not inhibit proteolysis of APP by BACE1 but instead prevent secretion of the cleavage products and lead to their accumulation within the cell. There is also evidence that GGA1 alters APP processing and transport independently of BACE1 [25], possibly in a SorLA/LR11-dependent manner [37, 38]. This might also explain the reduced sAPPα secretion upon GGA expression. Redirection of BACE1 and APP into the endo-lysosomal pathway will reduce the proteolytic processing of APP by α-secretase. Nonetheless, overexpression of APP, BACE1, and GGAs may lead to increased levels of the proteins in distinct compartments such as Golgi or endosomes, supporting enhanced cleavage activity and cleavage products.

To further address the question of whether APP processing can be influenced by a disturbance in BACE1-GGA interaction—similar to siRNA knockdowns—we used different GGA mutants. Interaction of BACE1 and GGA1, 2, and 3 via DISLL motif in the BACE1 C-term and VHS domain of all three GGAs was demonstrated previously [6, 33, 39, 40]. However, using full-length proteins, we saw that neither deletion (Fig 4) nor autoinhibition (S2 Fig) of the GGA1-VHS domain led to altered APP processing, as shown by ELISAs (Fig 4) and Western blot analysis (S2 Fig). More importantly, none of the mutants used was able to influence BACE1-GGA interaction in CoIP experiments (S2B Fig, Fig 4). These findings are in line with other studies in which deletion of the GGA1-VHS domain did not alter BACE1 maturation and generation or secretion of APP-β cleavage products [25]. Furthermore, BACE1 lysosomal degradation mediated by GGA3 is not altered upon mutation of the GGA3-VHS domain or the BACE1-DISLL motif [21]. The discrepancy in the results might be due to the different methods and approaches that have been used to study BACE1-GGA interaction. For CoIP experiments, we used full-length BACE1 and GGAs, whereas others [33] have used synthesized GST-VHS domains and BACE1 C-terms to performed isothermal titration calorimetry and GST-pull down experiments [6]. We do not exclude the possibility that BACE1 and GGA interact via VHS/DISLL domains. However, in this study, we provide strong evidence that the main functional interaction in APP processing between the full-length proteins is facilitated by GGA GAE domains.

The idea of GGA1 and GGA3 autoinhibition by an internal DXXLL motif has been discussed in different studies [22, 41]. Recently, this autoinhibition has been called into question [32]. Additionally, it was reported that a functional DXXLL motif must be located no more than one to three residues from the C-terminus of cargo proteins. Accordingly, autoinhibition mutants of GGA1 affected neither BACE1 interaction nor APP processing (S2 Fig) in our study. This might indicate that in living cells, autoinhibition does not play a role in the regulation of GGA1 activity. On the other hand, in our experiments, the addition of a HA-tag to the BACE1 C-terminus did not result in abrogated interaction with GGAs, as has been predicted. However, as we have shown that GGA-BACE1 interaction is VHS-domain independent, our results cannot sufficiently contribute to the discussion about GGA autoinhibition. Instead, they strengthen the idea of a GGA-VHS-domain-independent mechanism for BACE1 transport.

Additional evidence for the interaction between GGA GAE domains and BACE1 is provided by the influence of GAE domains on APP processing. Whereas overexpression of VHS deletion or autoinhibiting phosphorylation mutants enhances the effects of GGA_wt on sAPPβ or Aβ secretion, GAE-Δ-mutants showed increased levels of sAPP as well as Aβ (Fig 4). In particular, GGA2- Δ-VHS overexpression further decreased secretion of sAPPs and Aβ compared to GGA2_wt. GGA3 VHS depletion on the other hand does not alter secretion of APP cleavage products compared to GGA3_wt. In contrast to GGA2, GGA1 as well as GGA3 contain ubiquitin binding sites in their VHS domain. In particular for GGA3 an additional role in lysosomal transport dependent on this ubiquitin binding was reported [17]. Therefore deletion of the GGA3-VHS domain might impair clearance of APP and/or its cleavage products from endosomes to lysosomes. In contrast depletion of GGA2-VHS which is lacking ubiquitin binding motifs does not. However, the strongest impact was achieved by deletion of the GAT domain, which is in line with previous reports [25]. Kang and colleagues have further reported that for GGA3-mediated lysosomal degradation, ubiquitination of BACE1 is crucial. Therefore, they suggested the GAT domain—which contains ubiquitin binding sites—as the domain of interaction. In our CoIP experiments, we did not see altered binding between GGAs and BACE1 upon deletion of the GAT domain. As GGA GAT deletion mutants can no longer be recruited to membranes (S4 Fig) and thereby they lose their ability to bind and transport cargo at the Golgi, the question of whether GAT-ubiquitin binding plays a role in BACE1-GGA interaction cannot be addressed by overexpression of this mutant. GGA GAE deletion mutants, on the other hand, show a physiological distribution throughout the cell (S4 Fig). The GAE domain is responsible for binding of accessory proteins. However, the influence of GAE mutants on APP β-cleavage and on BACE1 interaction together provides strong evidence for a critical role of this domain in the transport and processing of APP by BACE1.

Similarity between the members of the GGA family in their ability to bind and transport BACE1 has been described previously [42]. In our experiments, all members of the GGA family showed similar effects on APP processing and interaction with BACE1. Furthermore, siRNA knockdown had the strongest effect on APP processing upon simultaneous knockdown of at least two GGAs (Fig 2). This fact indicates the ability of the GGAs to act together or to compensate for the reduction of one member of this protein family. Evidence that GGA homologues act cooperatively in sorting cargo was described previously [15, 43].

To analyze whether the GGAs show a difference in expression patterns supporting specific pathophysiological roles, we performed *in situ* hybridization, Western blot, and qPCR (Fig 5). Whereas we found lower mRNA levels of GGA2 compared with those of GGA1 and 3, GGA1 showed higher protein expression levels (Fig 5B). This might indicate different regulatory mechanisms for GGA expression and turnover. However, all GGAs are expressed in hippocampal neurons, but GGA2 is the only one expressed in astrocytes (Fig 5C). No data are available to date on the role of GGA2 in the brain. In human temporal lobe samples, we found increased GGA2 levels associated with increased GFAP levels (Fig 5D). This finding points to a specific role of GGA2 in astrocytes. APP processing by BACE1 and Aβ generation has been found in astrocytes, but whether they are a significant source of Aβ in AD is still a matter of debate [44, 45]. Levels of BACE1 in astrocytes are lower compared with those in neurons [2, 46]. However, astrocytes outnumber neurons by fivefold. Furthermore, a recent publication has shown that BACE1 and APP are upregulated in astrocytes upon stimulation with cytokines and Aβ, thereby increasing Aβ generation [47].

Whether GGA2 regulates BACE1 trafficking in astrocytes and can influence APP processing in these cells has to be addressed in further experiments. Furthermore, GGA2 and its mutants showed slightly different efficiencies in APP processing and BACE1 interaction compared with those of GGA1 and 3. Deletion of the GGA2 GAE domain had a smaller effect on BACE1 interaction, whereas deletion of the VHS domain enhanced binding to BACE1 (Fig 3). In line with this finding, deletion of the GGA2 VHS domain even increased the effects on sAPPβ and Aβ secretion, whereas the GAE deletion mutants had small effects compared with those of GGA1 and 3. This might indicate that GGA2 tends to bind cargo other than BACE1 via its VHS domain and that depletion of the VHS domain enhances the frequency of BACE1 interaction with the GAE domain.

**Fig 6:**
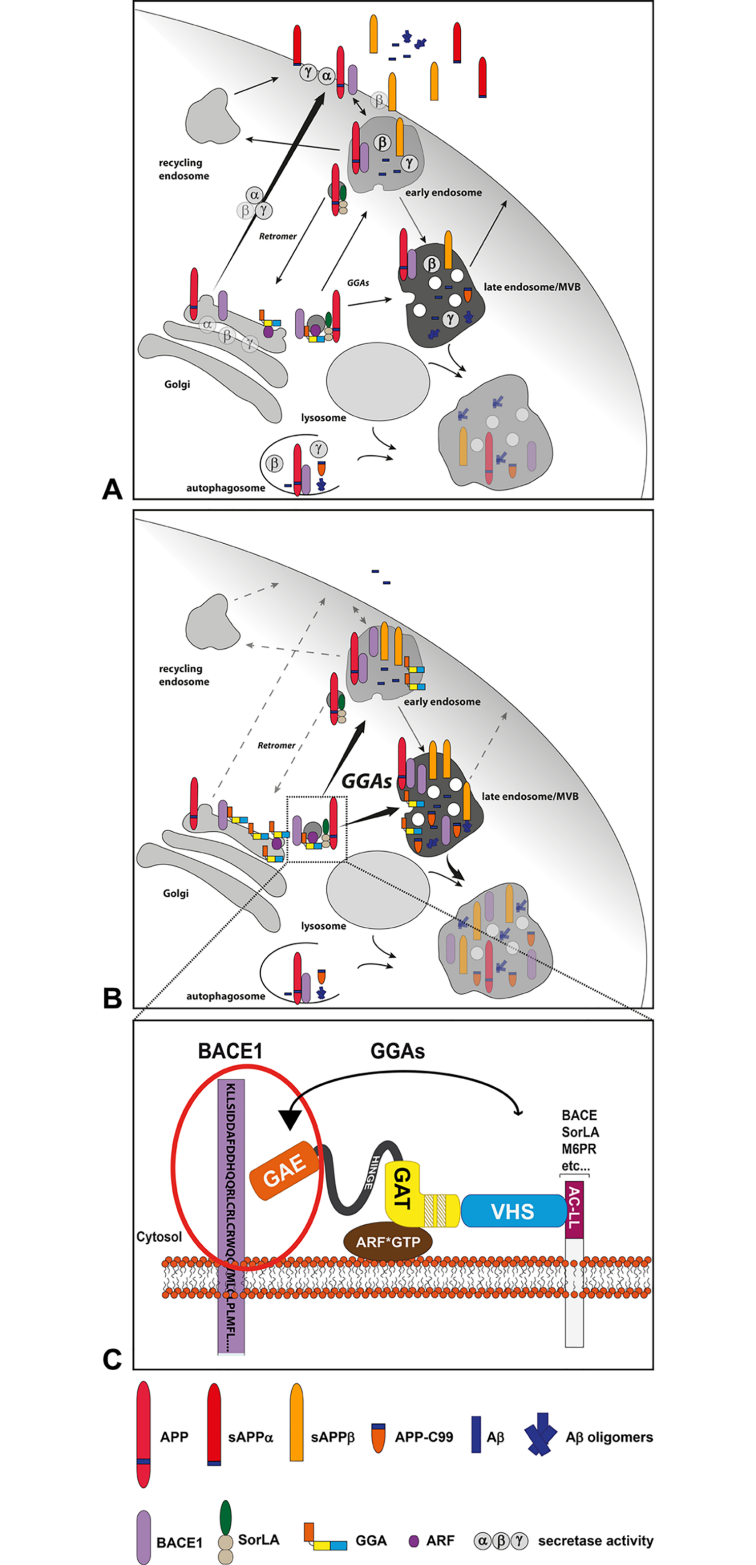
GGA-dependent BACE1 sorting and APP processing. **(A)** APP and BACE1 are transported to the cell surface via secretory pathways. Both can be internalized into endosomal compartments where acidic conditions promote APP cleavage by BACE1 and Aβ generation. APP cleavage products and BACE1 are either recycled to the cell surface or sorted into late endosomal/lysosomal parthway for degradation. Likewise, APP, C99 and β- and γ-secretase can be sorted into autophagosomes for degradation as reviewed [61]. Increased secretase activity in these compartments were described to enhance Aβ production and the acidic conditions also promote Aβ aggregation [62–64]. Therefore, disturbed lysosomal degradation leads to intracellular increase of Aβ and Aβ oligomers and might enhance the release into the extracellular space [65, 66]. (B) GGAs directly connect the TGN and the endosomal/lysosomal system [9–11] and thereby can bypass cell surface transport of BACE1 and APP-SorLA [37]. Increase of GGA levels result in increased levels of intracellular sAPPβ and decreased levels of extracellular sAPPs and Aβ indicating enhanced endosomal/lysosomal transport of BACE1 and APP. (C) Whereas, SorLA GGA interaction is dependent on GGAs VHS-domain and SorLAs DXXLL motif, interaction of GGAs and BACE1 is primarily mediated by GGAs GAE-domain.

In summary, our data support the model in which GGAs contribute to endosomal/lysosomal transport of BACE1 and maybe also APP (Fig 6). There is evidence that interaction of the GGA isoforms with and transport of BACE1 might contribute to the development of AD pathology. In addition, reduced levels of GGA1 and 3 have been found in AD patients [25, 34, 43, 48]. As GGAs influence BACE1 transport and appearance in endosomes, they are promising targets to modulate APP processing by BACE1 and thereby the pathological production of Aβ. Here we provide evidence for a novel interaction site of GGAs and BACE1, which offers the possibility to alter APP processing without influencing other GGA cargo such as SorLA.

## Supporting Information

S1 Fig GGA expression increases intracellular levels of sAPPβ and C99. N2A cells were co-transfected with APP and BACE and either GGA1, 2, or 3. Equal expression of all proteins was controlled by Western blot analysis. We found, significantly increased levels of sAPPβ in cell lysates upon GGA overexpression compared with control, as measured by ELISA. Furthermore, sAPP as well as C99 levels were increased in Western blot analysis. Shown are the results of n = 4 independent experiments. Statistical analysis was performed by using Kruskal-Wallis one-way analysis of variance (ANOVA) on ranks and multiple comparison (Dunn’s method) (*p<0.05).
(TIF)

S2 Fig Autoinhibition of the VHS domain does not influence APP processing or GGA1-BACE1 interaction. (A) Co-expression of APP, BACE1, and GGA1-wt, GGA1_S355A, GGA1_S355D, or control in N2a was controlled by Western blot. Experiments were carried out in triplicate. Neither the nonphosphorylatable (Lane 3) nor the pseudo-phosphorylated (Lane 4) GGA1 form altered APP processing compared with GGA1_wt, as shown by equal intracellular levels of sAPPs. (B) HA-tagged BACE1 was co-expressed with GGA1_wt-myc, GGA1-ΔVHS-myc, GGA1_S355A-myc, or GGA1_S355D-myc in HEK293. Equal expression was controlled by Western blot (left panel). BACE1 was immunoprecipitated by using anti-HA magnetic beads (Miltenyi Biotec). Co-precipitation of the GGAs was controlled by Western blot and anti-Myc antibody (9E10 (Sigma/M4439)). We observed no difference in the binding of BACE1 to GGA1-ΔVHS, GGA1_S355A, or GGA1_S355D compared with GGA1_wt (right panel).
(TIF)

S3 Fig SorLA-GGA1 interaction is dependent on VHS domain and "unblocked" DXXLL motif. (A) Myc-tagged GGA1-wt was precipitated from N2A lysates by using anti-Myc (9e10 (Sigma/M4439)) antibody labeled magnetic beads (Miltenyi Biotec) following the manufacturer’s protocol. Co-precipitated SorLA was visualized by Western blot and anti-SorLA antibody (BD/612633). Whereas non-tagged SorLA was co-precipitated by GGA1 (right panel, Lane 4), binding between SorLA and GGA1 was blocked upon addition of a GFP tag at the SorLA C-term (right panel, Lane 3). Equal expression of the proteins was ensured by Western blot analysis (left panel). (B) Using the same approach, we tested whether addition of an N-terminal GFP tag impairs the interaction of SorLA and GGA1. Addition of an N-terminal GFP tag did not impair GGA1 binding (right panel, Lane 2). (C) Myc-tagged GGA1-wt, -ΔVHS, and—ΔGAE were precipitated as described above. Compared with control (right panel, Lane 2), deletion of the GGA1 VHS domain impaired GGA1 and SorLA interaction, whereas deletion of the GAE domain had no impact on the binding capacity (right panel, Lane 4).
(TIF)

S4 Fig Deletion of the GAT domain leads to nonphysiological cytosolic distribution of GGAs. N2A cells were transfected with myc-tagged GGA_wt and domain deletion mutants. GGAs were immunostained by using anti-Myc antibody (9E10/Sigma) as the primary antibody and Alexa488 as the secondary antibody (Molecular Probes). Cells were analyzed by fluorescence microscopy (Zeiss Axiovert 200). Mutants with VHS or GAE domain deletion showed subcellular distribution similar to that of GGA_wt. However, deletion of the GAT domain of all three GGAs, which is responsible for membrane recruitment, led to an equal, nonphysiological distribution throughout the cell.
(TIF)

S5 Fig GGAs and BACE1 are differentially expressed in brains of postnatal rats. *In situ* hybridization of horizontal and sagittal sections of the developing rat brain shows the early postnatal expression of BACE1 as well as GGA1-3 with the highest levels at PD21. Strong signals can be detected in the cerebellum and in the hippocampus from PD9 onward, while PD3 sections show a uniform mRNA expression in the cortex, striatum, brain stem, cerebellum, and hippocampus. Besides these regions, BACE1 can also be detected in thalamic nuclei. Within the GGA family, GGA2 shows the strongest expression during all stages of early brain development.
(TIF)

S1 Table Human brain postmortem samples. Diagnosis of patients was based on combined neuropathological (plaque burden, Braak staging) and neuropsychological (CERAD) examinations. Listed are the patients of which post-mortem tissue was obtained for this study. (CAA = Cerebral Amyloid Angiopathy, CTRL TEST = Control with cognitive testing shortly before death, Dx = Diagnosis, NA = Not Available, PMI = Postmortem Interval).
(DOCX)
