# Supplementary figures and images for: The Golgi-Localized γ-Ear-Containing ARF-Binding (GGA) Proteins Alter Amyloid-β Precursor Protein (APP) Processing through Interaction of Their GAE Domain with the Beta-Site APP Cleaving Enzyme 1 (BACE1)
Source: PLoS One. 2015 Jun 8;10(6):e0129047. doi: 10.1371/journal.pone.0129047 (PMC4460050; doi:10.1371/journal.pone.0129047)

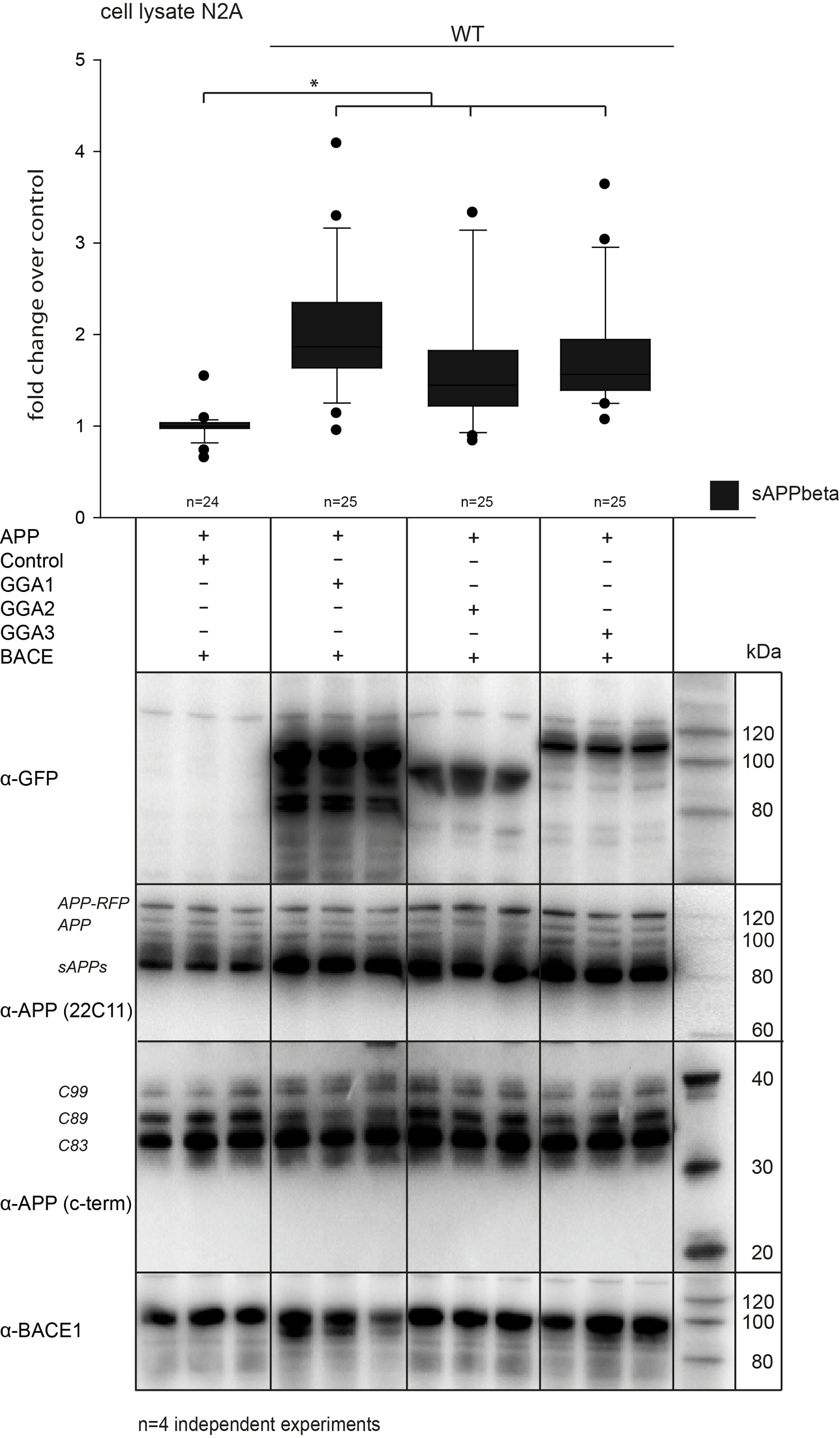

Supplement: S1 Fig — N2A cells were co-transfected with APP and BACE and either GGA1, 2, or 3. Equal expression of all proteins was controlled by Western blot analysis. We found, significantly increased levels of sAPPβ in cell lysates upon GGA overexpression compared with control, as measured by ELISA. Furthermore, sAPP as well as C99 levels were increased in Western blot analysis. Shown are the results of n = 4 independent experiments. Statistical analysis was performed by using Kruskal-Wallis one-way analysis of variance (ANOVA) on ranks and multiple comparison (Dunn’s method) (*p<0.05). (TIF) [file pone.0129047.s001.tif]

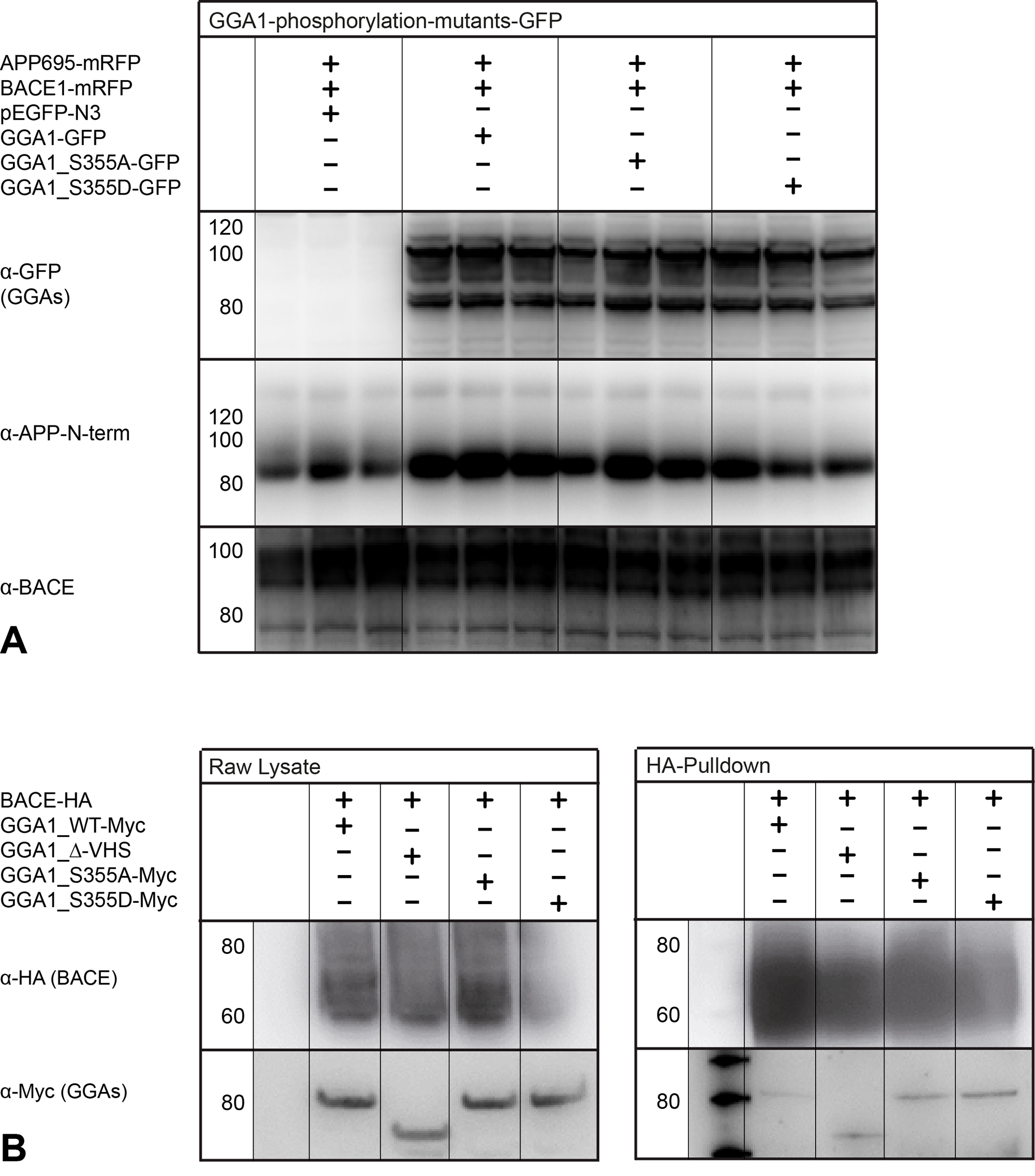

Supplement: S2 Fig — (A) Co-expression of APP, BACE1, and GGA1-wt, GGA1_S355A, GGA1_S355D, or control in N2a was controlled by Western blot. Experiments were carried out in triplicate. Neither the nonphosphorylatable (Lane 3) nor the pseudo-phosphorylated (Lane 4) GGA1 form altered APP processing compared with GGA1_wt, as shown by equal intracellular levels of sAPPs. (B) HA-tagged BACE1 was co-expressed with GGA1_wt-myc, GGA1-ΔVHS-myc, GGA1_S355A-myc, or GGA1_S355D-myc in HEK293. Equal expression was controlled by Western blot (left panel). BACE1 was immunoprecipitated by using anti-HA magnetic beads (Miltenyi Biotec). Co-precipitation of the GGAs was controlled by Western blot and anti-Myc antibody (9E10 (Sigma/M4439)). We observed no difference in the binding of BACE1 to GGA1-ΔVHS, GGA1_S355A, or GGA1_S355D compared with GGA1_wt (right panel). (TIF) [file pone.0129047.s002.tif]

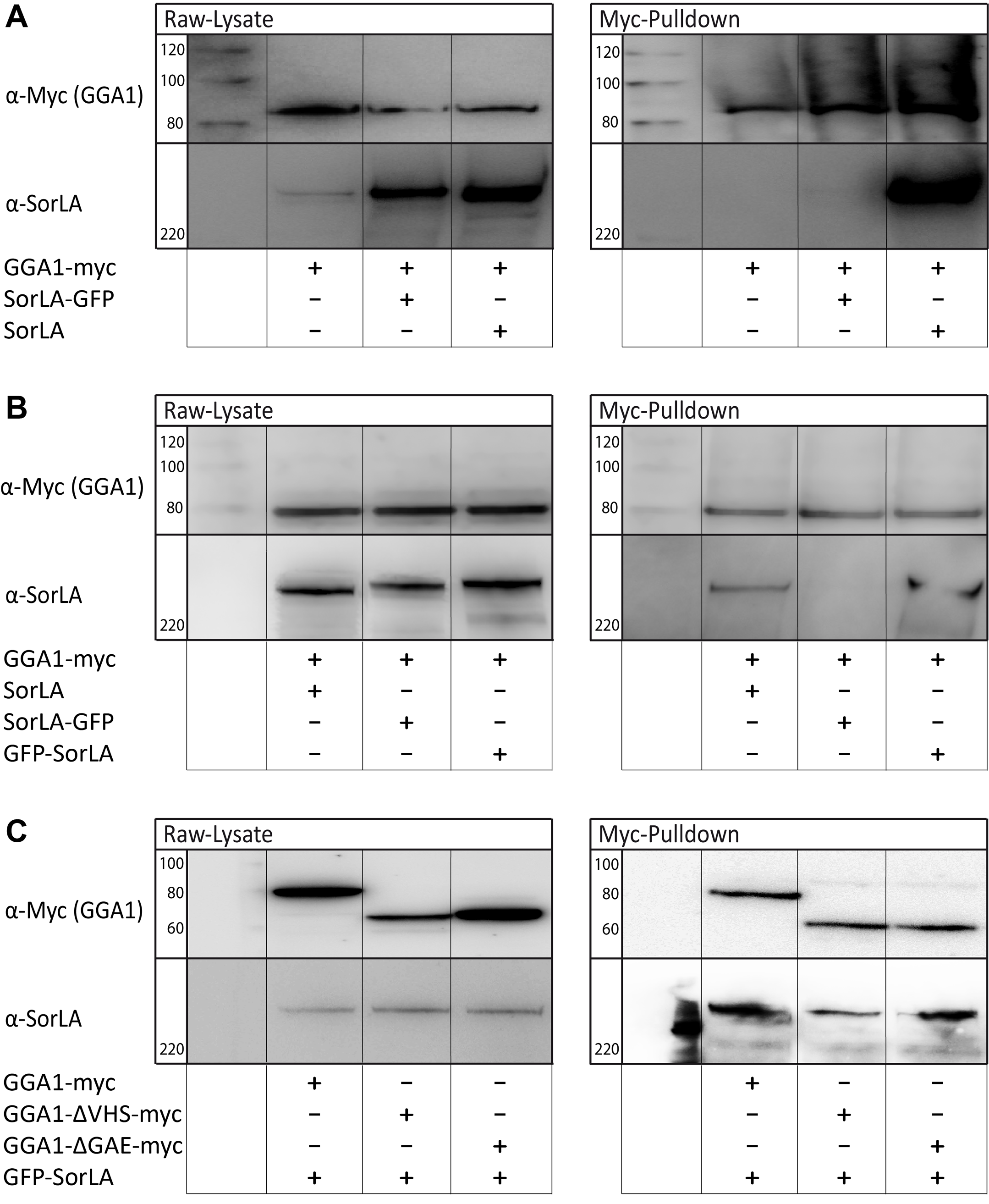

Supplement: S3 Fig — (A) Myc-tagged GGA1-wt was precipitated from N2A lysates by using anti-Myc (9e10 (Sigma/M4439)) antibody labeled magnetic beads (Miltenyi Biotec) following the manufacturer’s protocol. Co-precipitated SorLA was visualized by Western blot and anti-SorLA antibody (BD/612633). Whereas non-tagged SorLA was co-precipitated by GGA1 (right panel, Lane 4), binding between SorLA and GGA1 was blocked upon addition of a GFP tag at the SorLA C-term (right panel, Lane 3). Equal expression of the proteins was ensured by Western blot analysis (left panel). (B) Using the same approach, we tested whether addition of an N-terminal GFP tag impairs the interaction of SorLA and GGA1. Addition of an N-terminal GFP tag did not impair GGA1 binding (right panel, Lane 2). (C) Myc-tagged GGA1-wt, -ΔVHS, and—ΔGAE were precipitated as described above. Compared with control (right panel, Lane 2), deletion of the GGA1 VHS domain impaired GGA1 and SorLA interaction, whereas deletion of the GAE domain had no impact on the binding capacity (right panel, Lane 4). (TIF) [file pone.0129047.s003.tif]

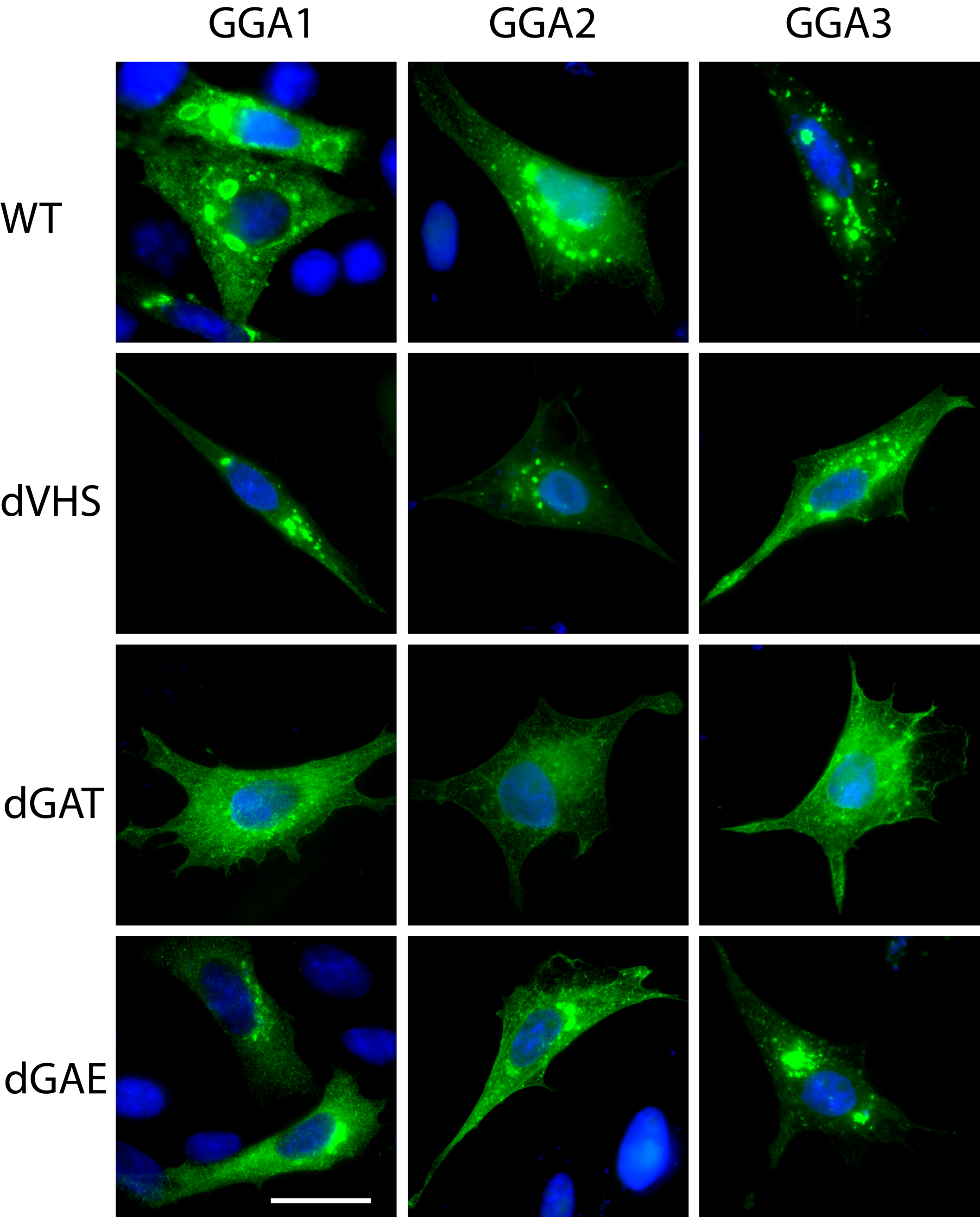

Supplement: S4 Fig — N2A cells were transfected with myc-tagged GGA_wt and domain deletion mutants. GGAs were immunostained by using anti-Myc antibody (9E10/Sigma) as the primary antibody and Alexa488 as the secondary antibody (Molecular Probes). Cells were analyzed by fluorescence microscopy (Zeiss Axiovert 200). Mutants with VHS or GAE domain deletion showed subcellular distribution similar to that of GGA_wt. However, deletion of the GAT domain of all three GGAs, which is responsible for membrane recruitment, led to an equal, nonphysiological distribution throughout the cell. (TIF) [file pone.0129047.s004.tif]

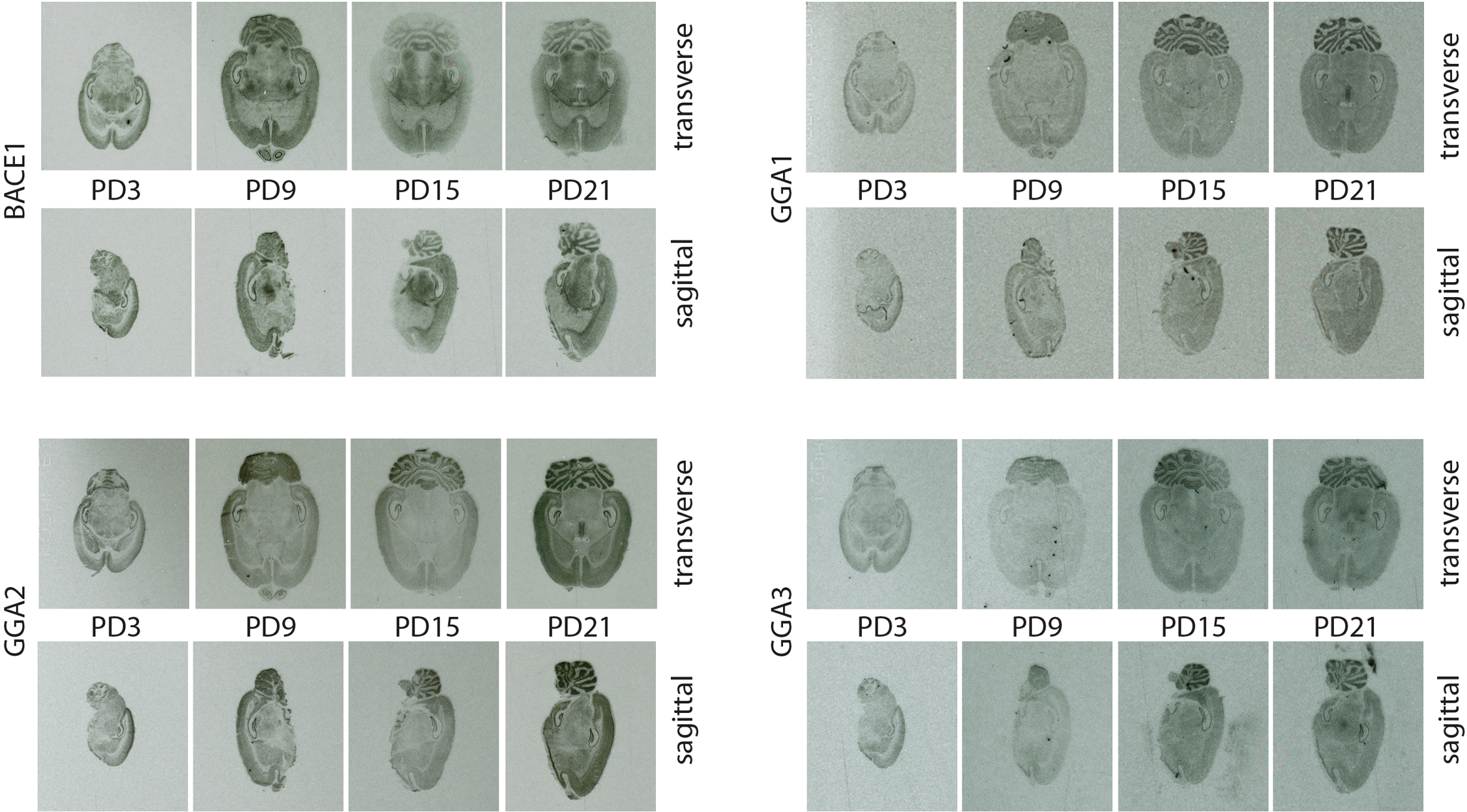

Supplement: S5 Fig — In situ hybridization of horizontal and sagittal sections of the developing rat brain shows the early postnatal expression of BACE1 as well as GGA1-3 with the highest levels at PD21. Strong signals can be detected in the cerebellum and in the hippocampus from PD9 onward, while PD3 sections show a uniform mRNA expression in the cortex, striatum, brain stem, cerebellum, and hippocampus. Besides these regions, BACE1 can also be detected in thalamic nuclei. Within the GGA family, GGA2 shows the strongest expression during all stages of early brain development. (TIF) [file pone.0129047.s005.tif]
