# Supplementary material for: The Golgi-Localized γ-Ear-Containing ARF-Binding (GGA) Proteins Alter Amyloid-β Precursor Protein (APP) Processing through Interaction of Their GAE Domain with the Beta-Site APP Cleaving Enzyme 1 (BACE1)
Source: PLoS One. 2015 Jun 8;10(6):e0129047. doi: 10.1371/journal.pone.0129047 (PMC4460050; doi:10.1371/journal.pone.0129047)
Supplement: S1 Table — Diagnosis of patients was based on combined neuropathological (plaque burden, Braak staging) and neuropsychological (CERAD) examinations. Listed are the patients of which post-mortem tissue was obtained for this study. (CAA = Cerebral Amyloid Angiopathy, CTRL TEST = Control with cognitive testing shortly before death, Dx = Diagnosis, NA = Not Available, PMI = Postmortem Interval). (DOCX) [file pone.0129047.s006.docx]

| **Clinical Dx** | **Neuropath Dx** | **Age at death (y)** | **Gender** | **Disease duration (y)** | **APOE genotype** | **PMI (h)** | **Plaque burden (%)** | **Braak stage** | **CERAD** |
| --- | --- | --- | --- | --- | --- | --- | --- | --- | --- |
| AD | AD | 85 | F | 4 | 3,4 | 10 | 2,086863 | NA | C |
| AD | AD | 88 | M | 10,29 | 3,3 | 4 | 2,259318 | NA | C |
| AD | AD | 84 | F | 7 | 3,3 | 7 | 2,02729 | NA | C |
| AD | AD | 86 | M | 16 | 3,4 | 12 | 3,051486 | NA | C |
| AD | AD | 92 | M | 22,03 | 4,4 | 12 | 1,768778 | NA | C |
| AD | AD | 74 | M | 11,29 | 3,3 | 15 | 3,135348 | NA | C |
| AD | AD | 84 | F | 15,54 | 3,4 | 12 | 4,318053 | NA | C |
| AD | AD | 77 | M | 6,39 | 4,4 | 18,5 | 2,555275 | NA | C |
| AD | AD | 78 | F | 6 | 3,4 | 8,5 | 2,712332 | NA | C |
| AD | AD | 80 | F | 12 | 4,4 | 10,5 | 4,966679 | NA | C |
| AD | AD | 81 | F | 9 | 3,4 | 3 | 2,719632 | NA | C |
| AD | AD | 82 | M | 6 | 3,4 | 7 | 3,900784 | VI | C |
| AD | AD | 93 | M | 17 | 3,3 | 6 | 1,892325 | V | A |
| AD | AD | 84 | M | 19 | 3,3 | 16 | 2,663821 | VI | A |
| AD | AD | 79 | M | 12,08 | 3,4 | 24 | 3,163545 | VI | C |
| AD | AD | 88 | M | 11 | 3,3 | 6 | 1,230971 | VI | B |
| CTRL | AD+CAA | 93 | M | 0 | NA | 14 | NA | VI | C |
| CTRL TEST | AD | 95 | F | 0 | 3,3 | 7 | 1,557704 | VI | B |
| AD | AD | 54 | F | 6 | 3,4 | 36 | 5,323695 | VI | C |
| CTRL | AD CHANGES | 86 | M | 0 | 3,3 | 20 | 1,840191 | II-III | B |
|  |  |  |  |  |  |  |  |  |  |
| CTRL | CTRL | 85 | M | 0 | 3,3 | 24 | 0 | II | No |
| CTRL | CTRL | 56 | M | 0 | 3,4 | 36 | 1,66542 | 0 | No |
| CTRL | CTRL, ACUTE HYPOXIA | 89 | F | 0 | NA | 13 | 0 | NA | NA |
| CTRL | CTRL | 76 | F | 0 | 3,3 | 24 | NA | I | No |
